# Case Report: High Prenatal Bisphenol A Exposure and Infant Neonatal Neurobehavior

**DOI:** 10.1289/ehp.1003064

**Published:** 2011-04-27

**Authors:** Sheela Sathyanarayana, Joe M. Braun, Kimberly Yolton, Stacey Liddy, Bruce P. Lanphear

**Affiliations:** 1 Department of Pediatrics, University of Washington, Seattle, Washington, USA; 2 Seattle Children’s Research Institute, Seattle, Washington, USA; 3 Department of Environmental Health, Harvard School of Public Health, Boston, Massachusetts, USA; 4 Department of Pediatrics, Division of General and Community Pediatrics, Cincinnati Children’s Hospital Medical Center, Cincinnati, Ohio, USA; 5 Child and Family Research Institute, British Columbia Children’s Hospital and Simon Fraser University, Vancouver, British Columbia, Canada

**Keywords:** bisphenol A, exposure, *in utero*, neurobehavior, prenatal

## Abstract

Context: Most of the U.S. population is exposed to the high-production-volume chemical bisphenol A (BPA), but targetable sources of exposure remain to be determined. Animal studies and one human study suggest that BPA is a neurotoxicant.

Case presentation: A mother in the Health Outcomes and Measures of the Environment (HOME) Study, a prospective birth cohort examining prenatal and postnatal environmental toxicants and childhood health outcomes, had a urinary BPA concentration of 583 µg/g creatinine at 27 weeks of pregnancy, which was the highest concentration observed in this cohort (median, 2.0 µg/g creatinine) and the general population. We used prenatal questionnaire data and a follow-up interview to identify potential sources of exposure that included daily plastic use and consumption of canned beverages and foods. Her male infant had a normal newborn neurobehavioral assessment but presented with abnormalities at the 1-month examination that prompted physician referral. Subsequently, the child had normal neurobehavioral testing results at annual evaluations from 1 to 5 years of age.

Discussion: Investigations into sources of high gestational urinary BPA concentrations provide an opportunity to identify potential targets for reduction of BPA exposure. This case highlights a potential link between gestational BPA exposure and transient neurobehavioral changes that is hypothesis generating and can serve to alert researchers to potential areas for examination in future studies.

Relevance to clinical practice: It is important to educate health care practitioners regarding potential sources of BPA exposure and anticipatory guidance on minimization of exposures during vulnerable periods of development.

Bisphenol A (BPA) is an estrogenic compound that was originally synthesized in the 1930s for pharmaceutical purposes (Dodds 1936). In the 1960s, the U.S. Food and Drug Administration approved alternative applications of BPA for use in hard polycarbonate plastics and, eventually, a wide variety of other consumer products, including dental sealants, food can linings, and water bottles (Food and Drug Administration 2010). Recent studies document that > 90% of the general population has measurable concentrations of urinary BPA, reflecting widespread exposure (Vandenberg et al. 2010). Although BPA has been identified in many commercial and industrial products, potential sources of BPA exposure in the general population still remain to be determined.

Because BPA is estrogenic, it has the potential to affect hormone-mediated neurologic and behavioral development in early life and is recognized as an endocrine-disrupting chemical. In 2008, a National Toxicology Program (NTP) expert panel identified BPA as having the potential to affect the developing fetal brain based on a number of animal studies reporting abnormalities in offspring after *in utero* exposures (Chapin et al. 2008).

The Health Outcomes and Measures of the Environment Study (HOME Study) is a prospective birth cohort study in Cincinnati, Ohio, that was designed to examine sources and exposures to a variety of prenatal and postnatal environmental toxicant exposures, including BPA, and subsequent health and neurobehavioral outcomes in children (Dietrich et al. 2005). The study obtained prenatal spot urine samples at approximately 16 and 26 weeks gestation and within 24 hr of delivery for assessment of maternal phthalate (chemicals used in plastics and personal care products) and BPA concentrations [Centers for Disease Control and Prevention (CDC) 2010]. Whole-blood and serum samples, collected from the mother at the same time points, were also analyzed for lead and cotinine, a metabolite of nicotine and biomarker of tobacco smoke exposure (CDC 2010). All laboratory analyses were performed by the National Center for Environmental Health at the CDC in Atlanta, Georgia (CDC 2010). Questionnaires on demographic and lifestyle characteristics were administered at approximately 20 weeks gestation, at birth, and at yearly childhood assessments from 1 to 5 years of age. Neurobehavioral assessments of infants and children were performed at birth, at 1 month of age, and then annually from 1 to 5 years using a full battery of validated psychometric tests.

This case study focuses on a mother in the HOME Study who had the highest reported BPA concentration in the cohort. Her male infant had a normal newborn examination followed by abnormal neurobehavioral findings on the Neonatal Intensive Care Unit Network Neurobehavioral Scale (NNNS) at 27 days of age. The NNNS is a comprehensive neurobehavioral assessment that assesses neurologic functioning, provides a behavioral profile, and measures signs of stress/abstinence in healthy and at-risk newborns. Summary scales allow for quantification along 13 dimensions of neurologic status and behavior. The stress/abstinence scale can be broken down into seven smaller subscales that describe specific organ systems (Lester and Tronick 2004).

*Ethical considerations.* The institutional review boards (IRBs) of the University of North Carolina–Chapel Hill, Cincinnati Children’s Hospital and Medical Center, the University of Washington, and the CDC approved this case study. The University of Cincinnati College of Medicine IRB was involved in the oversight of this study in the early stages of planning and implementation. All mothers provided written informed consent for themselves and their children before enrollment in the HOME Study. The mother of this case infant has also provided consent specific to the publication of this case report. The HOME Study reported the prenatal BPA and phthalate results to all participants for whom there was sufficient sample volume to conduct testing, and the handout from the Pediatric Environmental Health Specialty Unit (PEHSU) website [Association of Occupational and Environmental Clinics (AOEC) 2009] was also mailed to study participants. The case infant’s mother was individually counseled regarding her elevated prenatal BPA concentration and how to minimize BPA exposures.

## Case Presentation

The case mother was a 26-year-old African-American woman (case mother) with three reported past pregnancies and one living child; she had no significant past medical history and negative prenatal laboratory testing for hepatitis B, syphilis, group B streptococcus, and glucose intolerance. She had a 27-week urinary BPA concentration of 583 µg/g creatinine (1,250 µg/L). This was the highest BPA value recorded within the entire cohort (*n* = 389 participants and 1,100 urine samples) (Table 1). Her other prenatal urinary BPA concentrations at 16 weeks and a birth were at the 84th (4.1 µg/g) and 49th (1.9 µg/g) percentiles, respectively. Because of concerns that the 583 µg/g urinary BPA concentration could be a spurious finding, we followed up with the CDC National Environmental Health Laboratory. The CDC confirmed this result by reextracting the sample twice to conduct repeat testing. The values obtained were very close to the value originally reported and higher than the highest standard on the calibration curve. They also documented that most BPA in the urine was conjugated and therefore did not reflect external contamination. The case mother had concentrations of serum cotinine, blood lead, and urinary phthalates ranging from the 25th to 84th percentiles within the cohort (Table 1).

**Table 1:** Prenatal concentrations of urinary BPA, phthalate, and serum cotinine for the case infant’s mother compared with values in the full HOME Study cohort.

16 Weeks					26 Weeks					Birth				
Metabolite	Median (25th, 75th percentile)	Case value	Case percentile	Median (25th, 75th percentile)	Case value	Case percentile	Median (25th, 75th percentile)	Case value	Case percentile
BPA (µg/g creatinine)		1.7 (1.1, 3.0)		4.1		83.7		2.0 (1.3, 3.2)		583.0		100		2.0 (1.2, 3.0)		1.9		48.8
LMW phthalates (µM/g)*a*		11.8 (6.4, 26.1)		13.5		55.2		11.7 (6.0, 28.5)		19.5		65.7		11.2 (5.9, 22.7)		19.5		70.3
HMW phthalates (µM/g)*b*		3.6 (2.1, 6.7)		4.4		60.9		3.3 (2.1, 7.2)		2.1		27.3		10.5 (4.4, 22.4)		4.4		24.8
DEHP metabolites (µM/g)*c*		2.5 (1.4, 5.3)		3.6		63.5		2.4 (1.4, 5.1)		1.6		30.5		9.1 (3.2, 21.7)		4.3		31.2
DBP metabolites (µM/g)*d*		1.4 (0.9, 2.1)		1.8		67.4		1.4 (0.9, 2.3)		1.3		42.4		1.3 (0.7, 2.5)		0.7		24.5
Cotinine (ng/mL)		0.03 (< LOD, 0.2)		0.07		60.5		0.03 (< LOD, 0.2)		0.1		67.1		0.02 (< LOD, 0.2)		0.1		70.5
Lead (µg/dL)		0.7 (0.5, 0.9)		0.8		64		0.7 (0.5, 0.9)		0.6		43		0.8 (0.6, 1.0)		0.6		32
LOD, limit of detection. All phthalates are creatinine standardized. **a**Low-molecular-weight (LMW) phthalates were monobutyl phthalate, monoethyl phthalate, and monoisobutyl phthalate. **b**High-molecular-weight (HMW) phthalates were monobenzyl phthalate, mono(2-ethylhexyl) phthalate, mono(2-ethyl-5-carboxypentyl) phthalate, mono-(2-ethyl-5-hydroxyhexyl) phthalate, mono-(2-ethyl-5-oxohexyl) phthalate, and mono-(3-carboxypropyl) phthalate. **c**Di(2-ethylhexyl) phthalate (DEHP) metabolites were mono(2-ethyl-5-carboxypentyl) phthalate, mono-(2-ethyl-5-hydroxyhexyl) phthalate, mono-(2-ethyl-5-oxohexyl) phthalate, and mono(2-ethylhexyl) phthalate. **d**Dibutyl phthalate (DBP) metabolites were monobutyl phthalate and monoisobutyl phthalate.																		

The mother of our case infant is an African-American woman with > 12 years of education. She was unemployed throughout the pregnancy and living with her 4-year-old daughter during the time she was pregnant with the case infant. The Home Observation for the Measurement of the Environment inventory (HOME inventory) scale (Caldwell and Bradley 2003) score at the child’s first birthday was 31, which suggested a low quality and quantity of stimulation and support available in the home environment (Table 2).

**Table 2:** Demographic characteristics of the case mother–infant pair compared with the full cohort (*n* = 389).

Variable	Full cohort: women and children [*n* (%)]	Case mother
Child sex				
Female		208 (53.5)		
Male		181 (46.5)		X
Missing		0		
Maternal race				
White		237 (61.7)		
Nonwhite		147 (38.3)		X
Missing		5		
Maternal education (years)				
< 12		41 (10.7)		
12		54 (13.8)		
> 12		289 (74.5)		X
Missing		5		
Income category (US$ per year)				
< 40,000		153 (40.7)		X
40,000 to < 80,000		120 (31.9)		
≥ 80,000		103 (27.4)		
Missing		13		
Marital status				
Married		249 (64.8)		
Single		135 (35.2)		X
Missing		5		
Maternal age category (years)				
< 25		96 (24.7)		
25–34		231 (59.4)		X
≥ 35		62 (15.9)		
Missing		0		
HOME score category (12 months)				
< 35		51 (15.3)		X
35–39		64 (19.2)		
≥ 40		219 (65.6)		
Missing		55		
Beck Depression Inventory at 20 weeks				
Minimal or mild depression (0–19)		345 (92.0)		X
Moderate or severe depression (≥ 20)		30 (8.0)		
Missing		14		

The case male infant was born in 2004, at term (39 weeks of gestation) by spontaneous vaginal delivery without complications. Labor and delivery were unremarkable, with Apgar scores of 9/9 at 1 and 5 min of age. The infant had a normal course in the newborn nursery and a normal NNNS at 14 hr and was discharged from the hospital approximately 38 hr after birth.

At the 1-month neurobehavioral assessment, completed on the 27th day of life during a home visit, the infant displayed the following clinical signs or symptoms: hypertonicity in the trunk and upper and lower extremities, setting sun sign, tremors, cogwheel movements and athetoid finger posturing, high-pitch cry, and extreme irritability (Table 3). Compared with other infants in the HOME Study, this infant had high NNNS scores for excitability, lethargy, and stress abstinence (reflecting a higher level of the neurobehavioral dimension) and lower scores for regulation and quality of movement (Table 4). Within the stress abstinence subscales, autonomic stress, central nervous system stress, and state stress were elevated compared with the rest of the cohort. Two dimensions of the NNNS—attention and handling—could not be assessed because the extreme irritability of the infant prevented completion of key items. In addition, habituation could not be assessed because it requires the subject to be asleep, which is uncommon at the 1-month examination because most infants are awake and interactive; approximately 89% of infants in the HOME Study cohort could not be assessed for habituation for this reason. The neurologic signs and behaviors noted on the individual exam for the case infant were clearly abnormal. The infant was referred to his primary care physician for follow-up and further workup, if indicated. All other general physical examination findings were reported as normal in the infant’s medical records. The infant went on to have annual neurobehavioral assessments at 1–5 years of age that were within normal limits. All neurologic assessments were performed by trained examiners within the HOME Study who were blinded to prenatal biomonitoring results.

**Table 3:** Clinically significant results of NNNS exam for the case infant at 1 month of age.

Abnormal findings in case NNNS exam	Neurobehavioral symptoms
Displayed hypertonicity in trunk, neck, and upper/lower extremities		Excessive or above-normal muscle tone or tension; the infant’s musculature becomes “stiff” or rigid, and the infant shows marked resistance to passive movements.
Setting sun sign observed during first third of examination		Eyes point downward, pupils partially covered by lower eyelids, and sclera visible above the pupils.
Low-frequency/high-amplitude tremors and high-frequency/low-amplitude tremors		Tremors are rapid, rhythmic oscillation movements with a segmented quality.
Cogwheel movements		Movements are slower, coglike, jerky.
Athetoid posture of fingers observed at beginning of examination		Some of the fingers are fully flexed while others are simultaneously extended, simultaneous flexion of the elbow and rotation of the upper limb, or extension at the elbow with rotation of the wrist. Athetoid movements are slow, writhing changes from one athetoid posture to another.
High-pitch cry		Infant’s cry is high pitched at any time during the examination when the infant is in a sustained crying state.
Extreme irritability		Infant fusses or cries throughout the examination. The fuss/cry seems to be insulated in the sense that is seems to control the infant and determines the flow of the exam. This is the infant who is “at the mercy” of his or her fussiness. Crying is persistent and excessive. The infant cries to minimal as well as vigorous handling and maybe even without stimulation.


**Table 4:** Results of NNNS summary scores for the case infant at 1 month of age compared with mean values for the full cohort.

One-month NNNS value				
Cohort		
NNNS scale	Case	*n*	Mean ± SD
Attention		NA		336		5.40 ± 1.38
Arousal		5		336		4.18 ± 0.69
Regulation		4.07		352		5.53 ± 0.79
Handling		NA		348		0.45 ± 0.31
Quality of movement		3.83		355		4.81 ± 0.59
Excitability		8		335		2.44 ± 2.03
Lethargy		9		355		4.12 ± 1.76
Nonoptimal reflexes		2		355		3.97 ± 1.63
Asymmetry		1		355		1.23 ± 1.08
Hypertonia		0		355		0.04 ± 0.21
Hypotonia		0		355		0.28 ± 0.55
Stress abstinence total*a*		0.27		355		0.13 ± 0.05
Physiologic stress		0		355		0.03 ± 0.12
Autonomic stress		0.33		355		0.14 ± 0.15
CNS stress		0.5		355		0.17 ± 0.12
Skin stress		0		355		0.11 ± 0.11
Visual stress		0.23		335		0.15 ± 0.09
Gastrointestinal stress		2		355		1.97 ± 0.11
State stress		0.29		335		0.14 ± 0.11
Abbreviations: CNS, central nervous system; NA, not assessed. **a**The stress abstinence total reflects a summary of the seven subscales listed below.						

To determine potential sources of the elevated urinary BPA concentration and to follow up on the development of the child, we used the prenatal questionnaire (completed in 2004) and conducted a focused follow-up telephone interview with the mother in the fall of 2009. We developed interview questions based on the mother’s responses to prenatal questionnaires collected at 22 weeks gestation, with the goal of potentially identifying specific sources of BPA exposure in the fall of 2004 when the 26-week urine sample was collected. From the 2004 questionnaire, we learned that the mother was single and unemployed. Although she reported being a nonsmoker with no exposure to second-hand smoke during pregnancy, her serum cotinine values in fact indicated exposure to second-hand tobacco smoke. Her values were not high enough for her to be classified as a smoker and were consistent across the pregnancy. The mother did report occasionally drinking wine between the estimated date of conception and 15 weeks gestation, but the amount never exceeded one drink in a typical day. She reported no illicit drug use and took no medications or supplements during her pregnancy except for prenatal vitamins during the first trimester. She reported drinking five canned soda beverages per week in the month before completing her prenatal questionnaire at 22 weeks gestation. These beverages included a combination of Pepsi (excluding Pepsi One), Coca-Cola (excluding Diet Coke), Ruby Red, Storm, Big Red, Snapple flavored teas, and KMX. Her diet consisted of canned or frozen vegetables one to three times per week and fresh vegetables four to six times per week. Her diet also included fish, meat, and dairy and was generally high in fat, including approximately four fast-food meals per week.

At the 2009 follow-up interview, the mother reported eating canned ravioli (generic brand) every day during the period in which the 26-week urine sample was taken. Her daily exposure to plastics included heating and use of plastic food storage containers in the microwave and eating and drinking from plastic plates and cups. She used a municipal water supply and did not know what kind of plumbing was used in her home. She did not report being hospitalized or receiving any kind of inpatient medical care during her pregnancy. Except for a tooth extraction before 16 weeks of gestation, she had no other dental work performed for the duration of the pregnancy. She was unemployed throughout the pregnancy and living with her 4-year-old daughter during the time she was pregnant with the case infant. Her home was within 2 miles of a whiskey factory, but there were no other industrial buildings in close proximity. Regarding her child’s medical condition, she stated that her primary care physician followed her child with normal checkups and did not note any abnormal behavioral or developmental findings or other health problems. The mother reported the child was in school and performing well.

## Discussion

We report on a mother who had a prenatal urinary BPA concentration two orders of magnitude higher than the mean or median urinary BPA levels documented in previous biomonitoring studies. We identified multiple potential sources of BPA exposure that could be avoided during vulnerable periods of development, including canned beverages and foods. Her infant had normal neurobehavior at birth followed by abnormal arousal, regulation, quality of movement, excitability, and lethargy at 1 month of age. The temporal relationship of the extremely high prenatal BPA value with the transient abnormal neurobehavioral findings is hypothesis generating and raises the question about whether these findings reflect BPA toxicity. However, the abnormal neurobehavior at 1 month might have been a result of another etiology, given that the infant had normal findings at birth and from 1 to 5 years of age. This case highlights a potential association between gestational BPA exposure and transient neurodevelopmental findings that can alert researchers to potential areas for examination in future investigations and epidemiologic studies.

The mother’s BPA concentration at 27 weeks of pregnancy was higher than any reported in the peer-reviewed literature for a person in the general population (CDC 2010; Vandenberg et al. 2010). Consumption of contaminated food and beverages is thought to be the single largest contributor to BPA exposure in the general population (Vandenberg et al. 2010; von Goetz 2010). Recent data show that airborne BPA may also be an important source of exposure (Fu and Kawamura 2010). The case mother reported eating canned ravioli daily during her second and third trimesters of pregnancy and eating canned or frozen vegetables one to three times per week in her prenatal questionnaire. Food can linings may contain BPA that can leach into foods (Vandenberg et al. 2007). The degree of BPA leaching increases under acidic, basic, and high-temperature conditions. Canned beverages are also documented sources of BPA exposure (Cao et al. 2009). The case mother reported drinking five canned beverages per week in the month before the 22-week questionnaire. This overall consumption pattern could have contributed to her high exposure concentration. A recent study showed that replacing canned and packaged foods with fresh foods in one’s diet significantly lowered urinary BPA concentrations (Rudel et al. 2011). Consistently using and microwaving plastics (including plastic plates, cups, and food storage containers used to store and reheat foods) may have also led to the increased urinary BPA concentration. Hard polycarbonate plastic, cardboard food storage containers, and plastic stretch film can contain BPA (Vandenberg et al. 2007).

Although we were unable to identify a specific exposure that led to the isolated high BPA concentration early in the third trimester, the combination of reported exposures may have contributed to the higher BPA concentration. BPA concentrations can vary considerably during the day based on multiple exposures and within person based on individual metabolism. Mahalingaiah et al. (2008) found that single urine samples showed moderate sensitivity for predicting a pregnant woman’s exposure categorization over time, but it is possible that the case mother’s prenatal BPA concentration was a transient value that does not reflect exposures throughout the second and third trimesters of pregnancy. It is also possible that an unidentified source of BPA was the primary source of the elevated prenatal exposure concentration. Finally, recall bias may have affected answers to follow-up questions regarding prenatal exposures because the follow-up interview occurred 5 years after the infant was born. In 2004, we acquired data from the 22-week questionnaire on canned beverage consumption that asked about exposures in the month before completing the questionnaire. We did not have specific prenatal questionnaire data at the exact time when the 26-week urine sample was collected but did review responses from the 22-week questionnaire with the mother and confirmed reported lifestyle and dietary responses for the 26-week period.

Demographic factors can also be associated with overall exposure concentrations, and women from low socioeconomic backgrounds tend to have slightly higher urinary BPA concentrations than do women from higher socioeconomic strata (Braun et al. 2010). Although she was unemployed and had a low HOME inventory score, her demographic profile did not stand out in any particular manner from the rest of the enrolled women (Table 2).

Data on prenatal BPA exposures and potential health impacts in humans are limited. An earlier study from this cohort showed a positive association between prenatal urinary BPA concentrations at 16 weeks of pregnancy and increased externalizing behavior, representing acting out styles that are categorized on hyperactivity and aggression scales, in female children at 2 years of age (Braun et al. 2009b). The mother of the male case infant had a BPA concentration that was much higher than the median of the cohort and was therefore excluded from that analysis. The abnormalities in the case infant’s neurologic outcomes and behaviors at the 1-month examination were not observed in the overall cohort analysis for BPA exposure (data not shown). Ten singleton infants in the cohort were referred to a physician for abnormal NNNS findings after the 1-month examination. The geometric mean 26-week prenatal BPA concentrations for the 10 mothers of infants who were referred based on NNNS findings at the 1-month examination were higher than those for the mothers of 345 infants who were not referred (5.8 vs. 2.2 µg/g creatinine). The ratio of these values is 2.67 (95% confidence interval, 1.62–4.40). However, these results should be interpreted cautiously given the small number of referred infants.

Multiple animal studies cite neurologic abnormalities in offspring after *in utero* BPA exposures (Chapin et al. 2008; NTP 2008). In mice, high-dose (50 mg/kg) prenatal BPA exposures were associated with increased anxiety in male offspring compared with females, and Cox et al. (2010) postulated that this could be attributable to impacts on the dopaminergic or estrogen receptor-β pathways. Kawai et al. (2003) found that prenatal low-dose (2 ng/g and 20 ng/g) BPA exposures were associated with a transient increased aggression score in male rats at 8 weeks of age that resolved at 12 weeks and thereafter. This transient nature of the neurobehavioral changes is similar to that of our case infant, whose symptoms of increased hypertonicity, tremors, abnormal movements, and extreme irritability at 1 month of age resolved in later testing.

Other environmental chemicals and endocrine-disrupting compounds, such as phthalates, lead, mercury, polychlorinated biphenyls, and pesticides, can also play a role in neurodevelopment. In a multiethnic cohort study of newborns in New York City, third-trimester urinary concentrations of some phthalate metabolites were associated with decreased orientation and alertness scores in girls and improved motor performance in boys assessed with the Neonatal Behavioral Assessment Scale at 5 days of age (Engel et al. 2009). These exposures were also associated with elevated scores on conduct and aggression problem scales in children at 4 and 9 years of age (Engel et al. 2010). The same urinary phthalate metabolites were measured in this case study mother (Table 1) and were within the interquartile ranges of those reported for the women in the New York City cohort. Few animal and human studies examine prenatal exposure to mixtures of chemicals and subsequent neurodevelopmental health impacts. Therefore, it is difficult to know whether the mother’s exposures to other environmental chemicals may have played a role in the infant’s neurologic development.

The NNNS is a validated tool to assess neurobehavior during early infancy (Lester and Tronick 2004). It was originally designed to assess neurobehavioral effects of prenatal exposure to drugs of abuse and prematurity, but it can also be used to assess healthy and at-risk infants (Lester and Tronick 2004). The clinical significance of an abnormal NNNS exam in the neonatal period for functioning in later childhood has yet to be determined, but Liu et al. (2010) reported the NNNS to be highly predictive of behavior problems, school readiness, and intelligence through age 4.5 years in at-risk children. Additional studies are necessary to validate the predictive ability of the NNNS in representative samples. Neonatal neurobehavior can be quite variable depending on timing of the examination as well as environmental factors, but the 1-month assessment time has been used in many published studies and is a more stable time point for evaluating newborn neurobehavior (Xu et al. 2011). Normal physical examinations of newborn infants by medical providers quickly assess neurologic status by examining reflexes, tone, and general behavior but do not sensitively assess the specific of neurologic and behavioral factors that are assessed by the NNNS. Other infants within the HOME Study had abnormal neurologic examinations, but some of these mothers did not have elevated prenatal urinary BPA concentrations. These cases may have resulted from other etiologies of abnormal neurobehavior that have not yet been explored. The case infant displayed abnormal neurologic signs and behaviors at the 1-month examination, with no obvious etiology, which prompted us to conduct this case study. It is unclear how long these symptoms were present after the NNNS assessment. These abnormal findings were not noted by any other medical assessments performed by health care providers, including the primary medical doctor for the infant.

Other potential causes of abnormal neurobehavioral findings in an infant include brain injury due to hypoxic injury at birth, genetic causes, or some kind of postbirth neurologic insult (Paine 1961; Volpe 2008). The birth record documented a normal labor and delivery without insult, and there was no known family history of abnormal neurologic disease. Signs and symptoms of these types of insults can present immediately or take several days to manifest. Neurologic deficits with known etiology tend to persist past the neonatal period and take considerable time to resolve, if they resolve at all. The case infant had an isolated abnormal neurobehavioral examination at 1 month of age but without any apparent sequelae. No significant medical history that could have contributed to neurologic disease is noted in the medical record, and the mother stated that her infant had a normal course after discharge from the hospital. We did not identify an alternative etiology for the abnormal neurobehavioral findings from the medical record/questionnaire from 2004 or maternal interview in 2009.

This case study highlights a high prenatal urinary BPA concentration at 26-week gestation and an abnormal neurobehavioral examination at 1 month of age. It is likely that multiple sources of BPA exposure, which may have been avoided with appropriate education and resources, contributed to the mother’s elevated urinary concentration during pregnancy. This case raises the intriguing possibility that gestational BPA exposure may be associated with abnormal infant neurobehavior, because BPA is a suspected neurotoxicant that has elicited transient abnormal findings in toxicologic studies. Case reports or series of high-dose BPA exposures during fetal development offer a unique approach to study sources of BPA exposure and potential targets of toxicity. It is reassuring that the infant’s neurologic status was normal at follow-up, but the long-term health impacts of *in utero* BPA exposure remain to be determined by future studies that assess longitudinal developmental outcomes.

## Relevance to Clinical Practice

The result of this case study confirms previous studies documenting multiple sources of BPA exposure in humans. These findings can alert and inform health care practitioners that the general population can be exposed to high concentrations of BPA during gestation, a critical time for organ system and brain development. There is increasing concern regarding gestational exposure to endocrine-disrupting chemicals such as BPA because of evidence that environmental toxicants may be associated with an elevated risk for abnormal neurodevelopment (Braun et al. 2006, 2009a; Carpenter and Nevin 2009; Mendola et al. 2002). These conditions present significant economic and psychosocial public health burdens for our society.

In light of evidence supporting BPA and other environmental toxicants as potential human health hazards, health care providers should be prepared to learn about BPA and other environmental endocrine-disrupting chemicals and to appropriately counsel patients on how to minimize exposures. Health care providers can learn how to conduct an appropriate environmental history and assessment and to consider potential health impacts through a variety of resources (listed in Table 5). They can also consult with physicians professionally trained in environmental health to help address specific medical conditions that may be related to BPA and other endocrine-disrupting chemical exposures through university-based PEHSUs that consist of physicians and other environmental public health professionals (AOEC 2006). The PEHSUs have created handouts for health care providers and patients on chemicals in plastics, potential harmful effects, and how to avoid exposures (AOEC 2009). Industry can also help educate health care practitioners and consumers by providing information on products containing BPA and reduce exposures in manufacturing when possible.

**Table 5:** Evidence-based pediatric environmental health resources for health care practitioners.*a*

Organization/program	Description	Contact information	Funding source
Association of Occupational and Environmental Clinics: Pediatric Environmental Health Specialty Units (PEHSUs) (2006)		Made up of professionally trained environmental health experts, including physicians; provide evidence-based education and consultations to health care providers, state and local governments, and individual families		http://www.aoec.org/PEHSU.htm		U.S. Environmental Protection Agency, Centers for Disease Control and Prevention, Agency for Toxic Substances and Disease Registry
American Academy of Pediatrics: *Pediatric Environmental Health *handbook (Green Book) (2003)		Provides description and clinical guidelines for addressing common pediatric environmental health topics		https://www.nfaap.org/netforum/eweb/dynamicpage.aspx?site=nf.aap.org&webcode=aapbks_productdetail&key=17837ee5-f0fd-4486-9bcc-64f986b0f703		American Academy of Pediatrics
National Environmental Education Foundation: Pediatric Environmental History Initiative (2011)		Provides numerous resources on environmental education, including handouts on taking a pediatric environmental health history		http://www.neefusa.org/health/PEHI/index.htm		Chartered by Congress in 1990 under the National Environmental Education Act to advance environmental knowledge and action
Physicians for Social Responsibility: Pediatric Environmental Health Toolkit (2009)		Provides evidence-based environmental health tool kits for health care providers to use; health care providers can earn CME credit for taking the tool-kit course		http://www.psr.org/resources/pediatric-toolkit.html#what		Physicians for Social Responsibility, a not-for-profit 501(c)(3) advocacy organization that won the Nobel peace prize in 1985 and is funded by private individual donations as well as charitable group donations
CME, Continuing Medical Education. **a**Table adapted from J.M. Braun and R. Hauser (2011), “Bisphenol A and children’s health” (Curr Opin Pediatr 23(2):233–239), with permission from Lippincott, Walters, and Wilkins.						
